# Interaction of serum resistin and smoking behavior in predicting depression risk: a sex-sensitive analysis

**DOI:** 10.1186/s12888-026-08419-w

**Published:** 2026-07-29

**Authors:** Claudia von Zimmermann, Christian Weinland, Johannes Kornhuber, Bernd Lenz, Christiane Mühle

**Affiliations:** 1 Department of Psychiatry and Psychotherapy, Friedrich-Alexander University Erlangen-Nürnberg (FAU), Schwabachanlage 6, D-91054 Erlangen, Germany; 2 Department of Addictive Behavior and Addiction Medicine, Central Institute of Mental Health (CIMH), Medical Faculty Mannheim, Heidelberg University, Mannheim, Germany

**Keywords:** Smoking, Depression, Inflammation, Adipokines, Resistin, Metabolic imbalance, Masculine depression

## Abstract

**Background:**

Affective disorders often co-occur with behavioral risk factors like smoking, and chronic metabolic disorders like obesity or type 2 diabetes. One possible regulation factor in these comorbidities could be resistin, an adipokine involved in inflammation and insulin resistance. Here, we investigate these factors in depressed patients, in a sex-sensitive approach.

**Methods:**

We assessed depression symptoms in a sex-balanced cohort using the Beck Depression Inventory-II and the Male Depression Risk Scale. We further evaluated smoking behavior using self-report and the Fagerström Test for Nicotine Dependence. Resistin levels were measured in blood samples.

**Results:**

Higher resistin levels were associated with increased risk of major depressive disorder (MDD) in a sex- and smoking-dependent manner, with the strongest effect observed in female smokers and ex-smokers. No association was found between resistin and depression severity, while alcohol use emerged as the strongest predictor of masculine depression symptoms.

**Conclusion:**

This study demonstrates that resistin interacts with smoking and sex to increase MDD risk, particularly in female smokers/ex-smokers, highlighting the importance of preventive strategies targeting depression risk in women with comorbid risk behaviors such as smoking.

**Trial registration:**

German Clinical Trials Register ID DRKS00015291, registration date 2020-01-06, retrospectively registered.

**Supplementary Information:**

The online version contains supplementary material available at 10.1186/s12888-026-08419-w.

## Introduction

Major depressive disorder (MDD) is a highly prevalent disease with profound effects on individual well-being. It is frequently accompanied by metabolic comorbidities and an increased risk of cardiovascular disease [25]. Obesity, alongside depression, represents a leading cause of disability [18, 63] and shares overlapping mechanisms with affective disorders [5, 51]. Overweight, and especially obesity, elevates the risk for various chronic conditions, including cancer [42], type 2 diabetes mellitus [7], atherosclerosis [21], arthrosis [43], or chronic inflammatory conditions, such as asthma [39]. Similar associations are well known for depression [9, 49] and a bidirectional relationship between depression and obesity has been described [31].

Smoking is another critical factor in this context. Nicotine use often serves as a maladaptive strategy to cope with emotional distress [17]. Individuals with mental illness are twice as likely to use tobacco (WHO [62]). MDD is associated with a lower likelihood of smoking cessation and higher relapse rates [60]. In females, stress promotes tobacco use vulnerability [53], while smoking and stress are critical sex-specific markers for increased MDD risk in women [54].

Anxiety symptoms again can be both a risk factor for, and a consequence of, depression [23]. When they co-occur, they form a complex clinical phenotype with heightened somatic symptoms, and greater disease severity [16, 24, 52]. Comorbid anxiety exacerbates metabolic dysregulation [6]. In women, those effects may be particularly pronounced due to sex-specific hormonal influences [26].

A type of depression that is particularly associated with increased substance use, including smoking, is the so-called masculine depression [56]. This subtype is marked by increased externalizing behaviors, such as irritability, aggression, or risk-taking [37, 44]. Symptoms of this phenotype can be assessed using, for example, the Gotland Male Depression Scale (GMDS) [64], the Male Depression Risk Scale (MDRS-22) [44], and the Gender-Sensitive Depression Screening (GSDS) [34, 35]. Notably, masculine depression can occur not only in men but also in women, particularly in response to stress exposure [36].

Tobacco consumption is associated with systemic inflammation [11]. Further, a genetic predisposition to tobacco use is closely linked to an increased risk of developing metabolic syndrome [30]. Moreover, smoking may modulate adipokine profiles, including resistin levels [19, 40].

Growing evidence suggests that adipokines play a crucial role in the pathophysiology of various somatic and mental disorders, largely by promoting pro-inflammatory states [10]. These bioactive peptides are among others secreted primarily by adipose tissue and immune cells. Adipokines regulate physiological functions such as inflammation, metabolism, and insulin sensitivity [10, 27]. One of these adipokines is resistin, which was originally identified for its role in insulin resistance [50].

Smoking is positively associated with elevated resistin levels, with this effect depending on the specific haplotype in the resistin gene promoter region [20]. Additionally, prior research showed that smokers exhibit significantly higher resistin levels independent of body mass index (BMI) [19]. Mechanistically, it has been demonstrated in 3T3‑L1 adipocytes that nicotine promotes the release of resistin by modulating ATP-dependent potassium channels [2].

Resistin has been assumed to regulate symptoms of mood disorders [29]. A comparative study showed increased resistin levels in depressed patients compared to healthy controls [41] and a change of resistin values after electroconvulsive therapy in depressed patients has been described [14, 38]. In accordance with this, Weber-Hamann et al., [59] have identified a positive association between free cortisol concentrations and circulating resistin levels in patients with depression as well as a decrease in resistin concentrations in patients remitted from major depression.

The interplay between smoking, depression, and metabolic dysfunction may thus contribute to a vicious cycle of physiological and psychological burden. However, these results are inconsistent with other studies failing to detect robust differences after controlling for confounding factors such as metabolic syndrome [28].

Beyond behavioral factors like smoking, adipokine secretion is also significantly influenced by internal physiological drivers, particularly steroid hormones, which exert robust endocrine control over adipose tissue. Specifically, research utilizing ovariectomized rat models has shown that estrogen exerts a strong inhibitory effect on resistin expression across various fat deposits, suggesting a key role for this steroid hormone in suppressing resistin production [22]. On the other hand, it has been reported that estrogen increases resistin mRNA and secretion in murine 3T3‑L1 adipocytes [8]. These studies suggest that resistin regulation is highly context‑dependent, with estrogen modulating this adipokine in a complex, environment-specific manner.

While nicotine, estrogen, and resistin likely interact through overlapping hormonal and inflammatory pathways, direct triple-interaction studies are lacking; consequently, the impact of resistin on depression risk—particularly in relation to established risk factors such as smoking and sex—remains insufficiently explored.

### Aims of the study

Against this background, the present study aimed to examine the relationships between major depression, resistin, and smoking behavior using a sex-sensitive approach. We further investigated the association between smoking status and circulating resistin levels in patients with depression. In addition to classical depressive symptomatology, the study considered externalizing symptom patterns described in the context of masculine depression.

Our first hypothesis was that the three-way interaction between smoking status, resistin levels, and sex would be associated with changes in depression risk. We then hypothesized that, based on already known findings of higher resistin levels in women and in smokers, resistin levels would be associated with an increased risk of depression, particularly among female smokers. Additionally, we hypothesized that resistin levels would be associated with depression severity within the group of depressed patients. Furthermore, as an exploratory hypothesis, we examined whether resistin differs between patients with high versus low levels of masculine depression symptoms. All models were adjusted for age, C-reactive protein (CRP), alcohol use, and BMI. In this way, we also examined the contribution of additional biological markers (e.g., inflammatory markers) and behavioral factors (e.g., alcohol use).

By integrating psychological symptoms with behavioral and biological correlates, this study aims to contribute to a more differentiated understanding of biopsychosocial mechanisms in depression, with a particular focus on sex-specific pathophysiological pathways and risk profiles that may inform more targeted preventive and therapeutic strategies.

## Methods

### Sample population

**Fig. 1:**
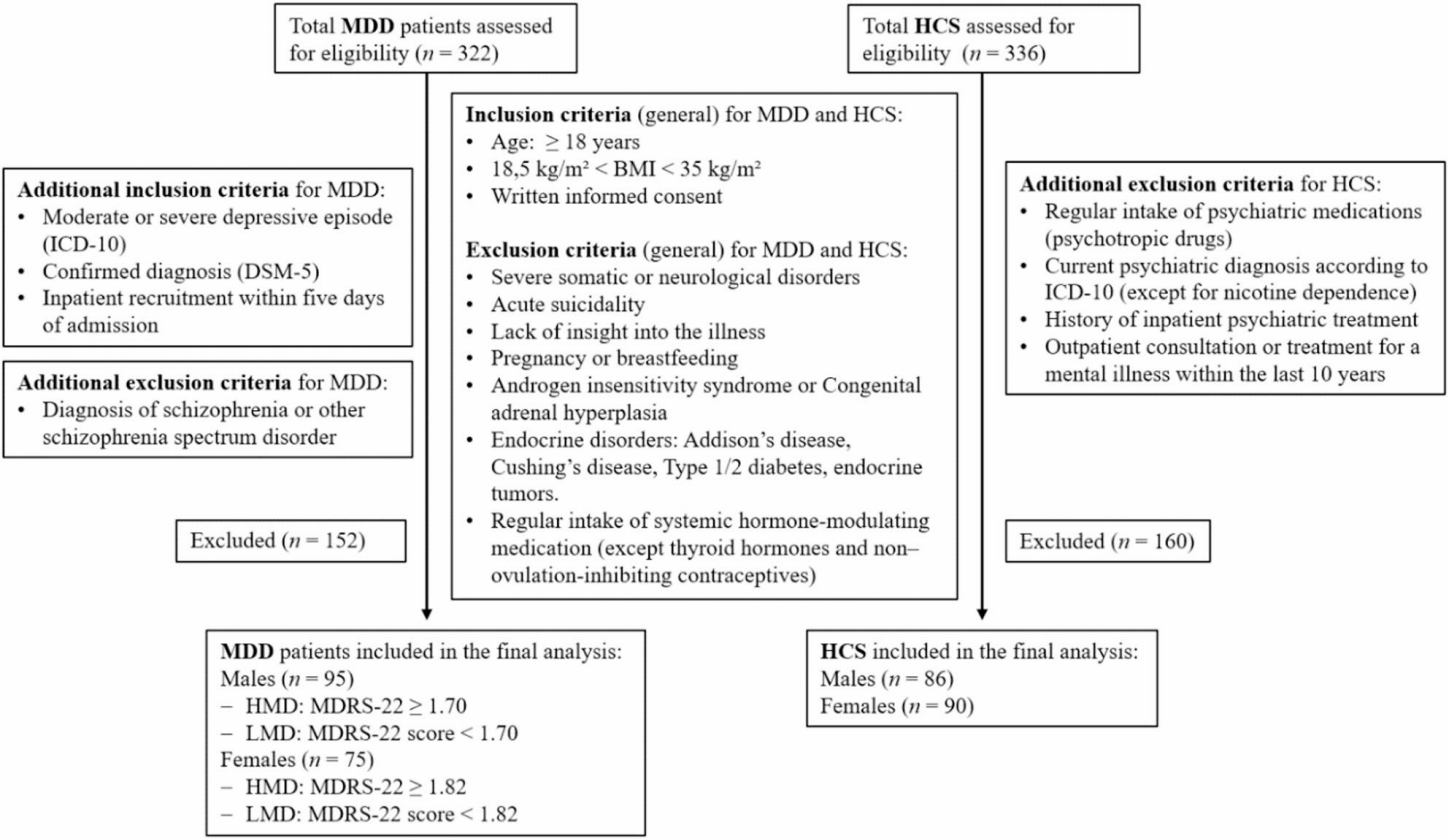
Flow diagram of participant recruitment. The diagram illustrates the screening of the initial 658 individuals, reasons for exclusion (*n* = 312), and the final inclusion of 346 participants. The specific focus on hormonal and endocrine stability in both groups were designed to align with the sex-related nature of the primary endpoints of the study. MDD Major Depressive Disorder, HCS Healthy Control Subjects, HMD High masculine depression scores, LMD Low masculine depression scores, MDRS-22 Male Depression Risk Scale

This study was designed as a prospective, open-label, comparative cohort study, with a single data collection point per participant, conducted as part of the Masculine Depression Project [47, 55–57]. Recruitment of participants took place from May 2017 to November 2019. A total of 658 individuals were screened, resulting in the inclusion of 170 patients—from the Department of Psychiatry and Psychotherapy at Friedrich-Alexander University Erlangen-Nürnberg (FAU) and the Clinic for Psychiatry, Addiction, Psychosomatics, and Psychotherapy at Europakanal in Erlangen, Germany—as well as 176 healthy control subjects (HCS). To ensure consistency, both groups were recruited from the same region and during the same time period by the same examiners. Each study visit lasted approximately 3 to 4 h and was conducted between 7 am and 10 am. General inclusion requirements were: a minimum age of 18 years, a BMI between 18.5 kg/m^2^ and 35 kg/m^2^, and written informed consent. Inclusion of patients required an inpatient stay due to a moderate or severe depressive episode according to ICD-10 in one of the two aforementioned clinics, or the presence of depressive symptoms consistent with a recurrent unipolar or bipolar affective disorder classified as moderate or severe under ICD-10 [61]. In addition, the diagnostic criteria for depression as defined by the DSM-5 [1] also had to be met. Study recruitment had to occur within the first five days of hospitalization. Participants were excluded if they had schizophrenia or other schizophrenia spectrum disorder. General exclusion criteria for patients with MDD and HCS were severe somatic or neurological disorders, acute suicidality, lack of insight into the illness, pregnancy or breastfeeding, androgen insensitivity syndrome or congenital adrenal hyperplasia, endocrine disorders, regular intake of systemic hormone modulators (except thyroid hormones and contraceptives that do not inhibit ovulation). HCS were recruited from the general community through flyers and social media platforms. Individuals expressing interest in the study underwent an initial telephone screening. Additional exclusion criteria for control subjects included regular intake of psychiatric medications (psychotropic drugs), current psychiatric diagnosis according to ICD-10 (except for nicotine dependence), history of inpatient psychiatric treatment, and outpatient consultation or treatment for a mental illness within the last 10 years. The overall selection process and the specific focus on hormonal and endocrine stability in both groups were designed to align with the sex-related nature of the primary endpoints of the study. Control subjects received a compensation of 30 euros for their participation. A flowchart of the recruitment process is provided in Fig. 1.

### Smoking behavior and alcohol consumption

We determined the smoking behavior by self-report (smoking, ex-smoking, never-smoking) and nicotine dependence using the Fagerström Test for Nicotine Dependence (FTND), with higher scores indicating greater nicotine dependence [15]. Alcohol consumption was assessed using the Alcohol Use Disorders Identification Test (AUDIT) in the German version [46].

### Evaluation of depression symptoms

Depression severity was assessed using the Beck Depression Inventory-II (BDI-II) [3]. The MDRS-22 was used to capture the characteristics of masculine depression. The scale comprises 22 items focusing primarily on externalizing symptoms considered characteristic of masculine depression and refers to experiences within the past month. Due to the absence of a German version at the time of recruitment, our study team translated the questionnaire developed by Rice et al., [44] into German. This process involved a forward–backward translation procedure with three independent bilingual translators, followed by reconciliation and pilot testing within the study cohort. The applied items are described in detail in our previous studies [56, 57].

To categorize participants into groups with high versus low masculine depression scores, a sex-specific median split was applied. Men with scores ≥ 1.70 and women with scores ≥ 1.82 were classified as having high masculine depression scores (HMD), while the others were classified as having low masculine depression scores (LMD). This data-driven approach was chosen to account for the lack of established clinical thresholds for the MDRS-22 in female populations and the specific nature of our inpatient sample, where all participants were already diagnosed with depression according to ICD-10 [61]. Notably, these sample-specific medians (1.60 and 1.80) align with or exceed the ‘elevated risk’ threshold (total score ≥ 32 points, corresponding to an item mean of ≈ 1.45) established in the original validation study by Rice et al., [45]. By using these cut-offs, we ensured that the HMD group represents a clinically relevant phenotype within an already severely burdened collective, rather than applying general population screening thresholds which might lack sensitivity in an inpatient setting.

### Blood samples and laboratory analyses

Subjects provided 346 blood samples, which were taken in the morning between 7 am and 10 am; 15 additional blood drawings took place until 12:15 pm. CRP levels were quantified at the Central Laboratory of the University Hospital Erlangen, Germany (DIN EN ISO 15189 accredited) within one day after blood collection. Resistin serum levels were determined in duplicates of 2 µl using the Human Resistin DuoSet ELISA (# DY1359, R&Dsystems, bio-techne GmbH, Wiesbaden, Germany) sandwich ELISA according to the manufacturer’s instructions with intra-assay and inter-assay coefficients of variation of 3% and 15%, respectively, and a standard curve ranging from 18 to 1600 pg/ml.

### Body measurement

Body height was assessed via self-report. Body weight (kg), body fat (%), muscle mass (%), resting metabolic rate (kcal), and visceral fat (%) were measured using bioelectrical impedance analysis scales (OMRON). Measurements were conducted under standardized conditions with participants barefoot and wearing light clothing. BMI (kg/m²) was calculated automatically by the device after entering the reported height.

### Statistical analyses

We used SPSS for Windows 29.0.1.0 (SPSS Inc., Chicago, IL, USA) to analyze the data. We used GraphPad Prism 8.4.3 (Graph Pad Software Inc., San Diego, CA, USA) and Dawson’s Excel template for three‑way interactions in binary logistic regression [12, 13] to visualize the results.

Descriptive analyses: Variation in categorical variables was tested using χ² tests, whereas continuous variables were compared using Mann–Whitney U tests. We employed Student’s t-tests for differences in two independent groups, and the statistics were adjusted when necessary, according to the Levene’s test. Correlations were analyzed using the Pearson method.

#### Primary analyses

Binary logistic regression analyses were conducted to examine depression status (depressed patients vs. healthy controls) as the binary outcome, with resistin, smoking status (smoking and ex-smoking vs. never-smoking), and sex as predictors. All models were adjusted for age, CRP, alcohol use (using AUDIT sum scores), and BMI. To ensure that the assumptions for binary logistic regression were met, multicollinearity among individual predictors was assessed using pairwise Pearson correlations; all coefficients fell within the generally accepted range of -0.5 to 0.5. A hierarchical regression analysis was conducted to examine the three-way interaction on depression risk. The models were built as follows: Model 3 included the main effects of resistin, smoking, and sex. Model 2 was extended by adding the three possible two-way interaction terms (resistin × smoking, resistin × sex, smoking × sex). Finally, Model 1 comprised all terms from the previous steps and additionally included the three-way interaction term (resistin × smoking × sex).

For the visualization of the results (Fig. 3), predicted probabilities were calculated using mean-centered continuous variables, with the mean for resistin set to 0. These values were then plotted using Dawson’s Excel template [13], displaying moderator combinations at low/high levels (smoking status: 0/1; sex: 0/1; resistin at 0 ± 1 SD).

Secondary and post-hoc analyses: To decompose significant higher-order interactions and investigate the specific risk associated with different factor combinations, post-hoc comparisons were performed. These included stratified binary logistic regression models (adjusted for the aforementioned covariates) to probe the effect of resistin on depression risk within specific subgroups (e.g., female smokers and ex-smokers).

Severity analysis: Within the subgroup of depressed patients, multiple linear regression models were used to test whether resistin levels predicted depression severity (assessed via BDI-II), controlling for age, sex, BMI, CRP, and AUDIT sum scores.

Exploratory analyses: Binary logistic regression models were conducted with high vs. low masculine depression scores (based on MDRS-22 sex-specific median cut-offs: ≥ 1.70 in males, ≥ 1.82 in females) as the dependent variable. Predictors included resistin, smoking status (smoking and ex-smoking vs. never-smoking), and sex. Age, BMI, CRP and AUDIT sum scores were entered as covariates.

Unstandardized coefficients (B) were reported. Bonferroni correction was specified for the primary hypothesis (three-way interaction), but no adjustment was required because only a single test was performed; secondary and exploratory analyses were uncorrected. Statistical significance was set at two-tailed *p* < 0.05.

## Results

### Cohort characteristics

**Fig. 2:**
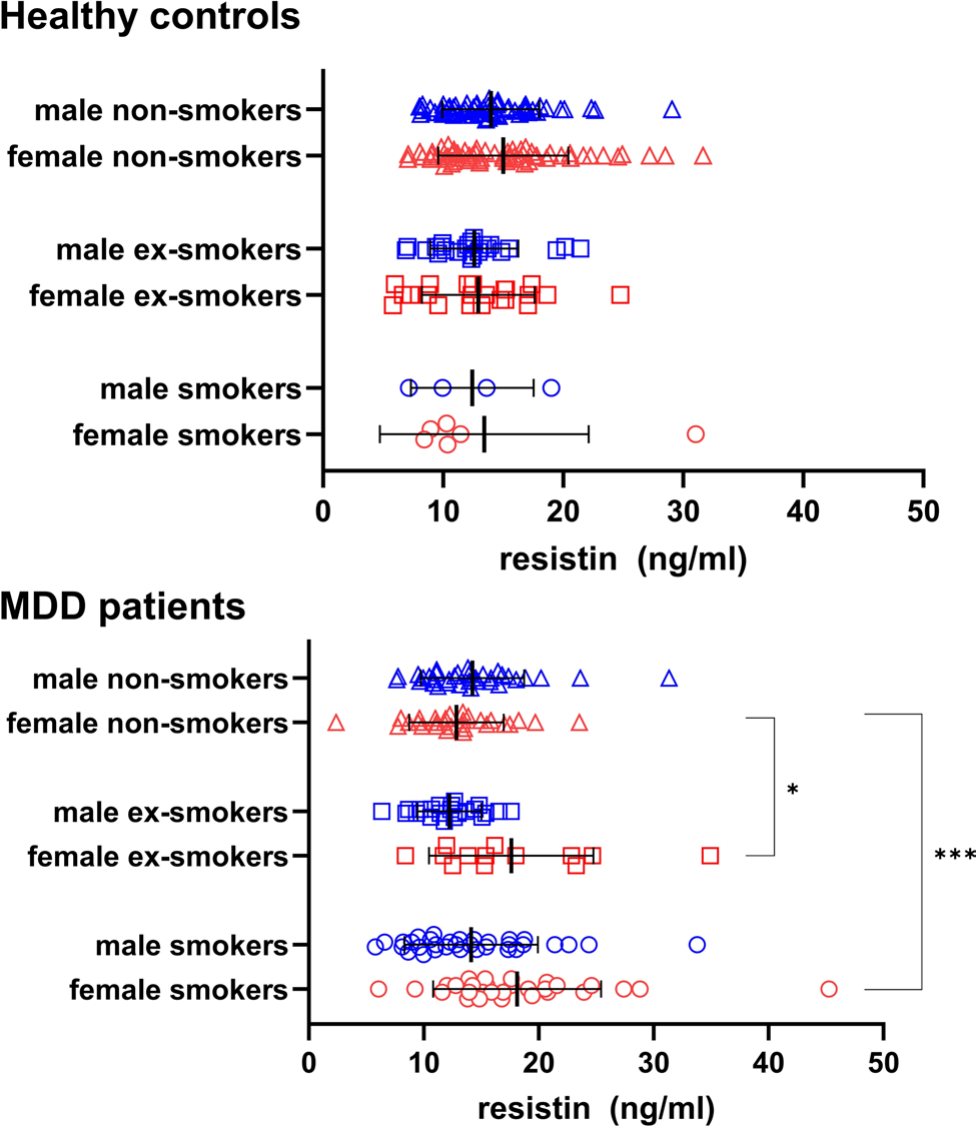
The figure illustrates the unadjusted raw data for resistin levels across the study groups, with error bars representing the mean and standard deviation (SD). MDD: Major Depressive Disorder. Statistical significance was determined using independent t-tests (* p < 0.05, *** p < 0.001)

Table 1 shows a sociodemographic comparison between patients with MDD and the HCS. Relative to the HCS, the depressed patients were older (mean age: 42 vs. 37 years), and more often current smokers (38 vs. 6 %). We found higher resistin values in female depressed smokers in comparison to female depressed non-smokers (U = 192.00, *p* < 0.001) within the group of depressed patients (Fig. 2). For results concerning the two patient subgroups — patients with HMD vs. LMD scores — please refer to Supplementary Table 1. Among patients, median MDRS-22 scores were 1.60 (IQR 1.17–2.58) in men and 1.80 (IQR 1.17–2.28) in women, with no statistically significant sex difference (Mann–Whitney test, *p* = 0.912). Based on these distributions, patients were categorized into a low masculine depression (LMD) group with MDRS-22 scores below the sex-specific cutoffs and a high masculine depression (HMD) group with scores at or above these thresholds. Men with scores ≥ 1.70 and women with scores ≥ 1.82 were classified as having HMD. The internal consistency of the MDRS-22 was good in both study groups, with Cronbach’s alpha values of 0.83 in patients and 0.79 in controls.

**Table 1:** Cohort characteristics

	MDD		HCS		MDD vs. HCS	
	*N*	M/F ± SD	*N*	M/F ± SD	χ² or t	*P*
Men	170	55.9 (%)	175	48.6 (%)	1.85	0.174
Smokers	162	38.3 (%)	175	5.7 (%)	52.71	**< 0.001**
Smokers and ex-smokers	162	58.8 (%)	175	32.6 (%)	26.83	**< 0.001**
Ex-smokers	162	20.6 (%)	175	26.9 (%)	1.33	0.249
Age (years)	170	41.75 ± 15.24	175	37.09 ± 13.72	2.99	**0.003**
BDI-II score	161	33.01 ± 11.25	173	3.38 ± 3.83	31.73	**< 0.001**
MDRS-22 score	163	1.89 ± 0.97	175	0.38 ± 0.39	18.49	**< 0.001**
FTND score	162	1.47 ± 2.46	174	0.06 ± 0.40	7.23	**< 0.001**
AUDIT score	160	6.31 ± 7.65	175	4.37 ± 3.06	2.99	**0.003**
CRP (mg/l)	170	3.35 ± 7.67	175	2.06 ± 6.06	1.74	0.083
LDL cholesterol (mg/dl)	170	135.01 ± 40.39	175	125.77 ± 33.57	2.31	**0.022**
Triglycerides (mg/dl)	170	136.75 ± 83.49	175	117.17 ± 76.34	2.27	**0.024**
Resistin (ng/ml)	170	14.94 ± 5.96	175	13.96 ± 4.82	1.68	0.094
BMI (kg/m^2^)	168	26.04 ± 4.24	174	25.00 ± 3.44	2.50	**0.013**
Body fat (%)	163	29.09 ± 9.36	174	27.74 ± 8.57	1.37	0.171
Body muscle (%)	162	31.92 ± 5.56	174	32.55 ± 5.69	-1.03	0.303
Visceral fat (%)	163	8.21 ± 4.13	174	6.95 ± 3.60	2.99	**0.003**

The table shows the valid number of subjects analyzed (N), means (M), or relative frequencies (F), standard deviation (SD), and the results of χ² and student’s t-tests. Participants with missing values for resistin were excluded from the statistical analyses. MDD Major Depressive Disorder, HCS Healthy Control Subjects, BDI-II Beck Depression Inventory-II, MDRS-22 Male Depression Risk Scale, FTND Fagerström Test for Nicotine Dependence, AUDIT Alcohol Use Disorders Identification Test, CRP C-reactive protein, LDL Low-density lipoprotein, BMI Body-Mass-Index. *P* < 0.05 in bold

### Association of smoking and resistin with risk of MDD

#### Primary three-way interaction analyses

**Fig. 3:**
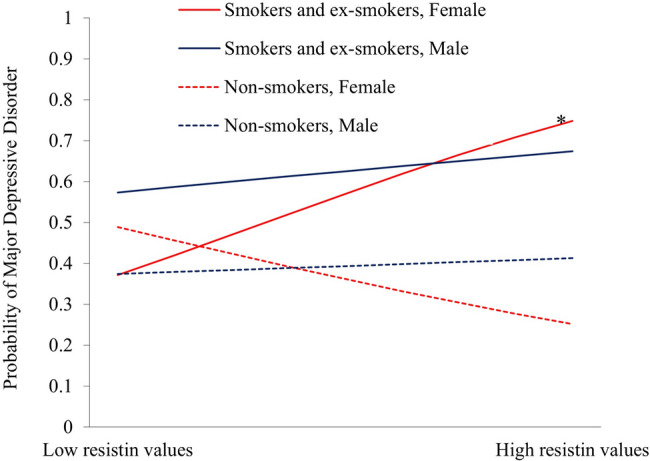
The figure depicts predicted probabilities of the binary outcome from the logistic regression model including the three-way interaction between resistin (independent variable), smoking status (first moderator), and sex (second moderator) using age, BMI, CRP und AUDIT as covariates. All continuous predictors, moderators, and covariates were mean-centered prior to analysis, and interaction terms were calculated using these mean-centered variables. Predicted values were calculated from the unstandardized coefficients and plotting lines for non‑smokers versus smokers/ex‑smokers and males versus females. The figure was created with Dawson’s Excel template for three‑way interactions in binary logistic regression [12, 13]. * indicates significant resistin effects (p < 0.05) from sex- and smoking status-stratified post-hoc logistic regression analyses adjusted for age, BMI, CRP, and AUDIT score (see Table 3 for details). BMI Body Mass Index, CRP C-reactive protein, AUDIT Alcohol Use Disorders Identification Test

Binary logistic regression analyses (*N* = 346) differentiated patients with MDD from HCS. The hierarchical model revealed a significant three-way interaction between resistin, smoking status (smoking and ex-smoking vs. non-smoking), and sex (female vs. male) on depression risk (B = 0.220, *p* = 0.040; Table 2, Model 1). The resistin × smoking two-way interaction (Model 2) was also significant (B = 0.145, *p* = 0.006). Among covariates in the model without interactions, smoking status (*p* < 0.001) and AUDIT sum score (*p* = 0.014) reached significance (Table 2, Model 3). As illustrated in Fig. 3, the effect of resistin on MDD probability was most pronounced among the group of female smokers and ex-smokers, showing a positive slope. In a sensitivity analysis excluding ex-smokers, the pattern of effects remained comparable but the interaction terms were no longer statistically significant, likely due to the loss of the intermediate exposure group and the associated reduction in statistical power.

#### Post-hoc stratified analyses

Following the significant three-way interaction, post-hoc binary logistic regression analyses stratified by smoking status and sex were conducted to probe group-specific effects of resistin on depression risk, adjusted for age, BMI, CRP, and AUDIT score (Table 3). Among female smokers/ex-smokers (*N* = 70), resistin emerged as a significant predictor of MDD vs. HCS group status (B = 0.146, *p* = 0.012). No significant resistin effects were observed in male smokers/ex-smokers, male non-smokers, or female non-smokers. Among covariates, age showed a significant effect in male non-smokers (*p* = 0.020), CRP and AUDIT sum score in male smokers and ex-smokers (*p* = 0.025 and *p* = 0.026).

### Association of resistin with depression severity

Within depressed patients, linear regression analyses examined the association between circulating resistin levels and depression severity (BDI-II scores), stratified by smoking status and sex, adjusted for age, BMI, CRP, and AUDIT score (Table 4). No significant associations emerged across any subgroups. None of the covariates reached statistical significance.

### High vs. low masculine depression scores

In a logistic regression model predicting HMD vs. LMD (HMD defined as MDRS-22 scores ≥ 1.70 in men and ≥ 1.82 in women), the AUDIT sum score was the strongest predictor (*p* < 0.001), followed by smoking status (*p* = 0.026) and age (*p* = 0.019). CRP, resistin, BMI, and sex were not significant predictors (Supplementary Table S2).

**Table 2:** Binary logistic regression to differentiate between patients’ groups and healthy controls with resistin as primary predictor and age, BMI, CRP, AUDIT score, smoking, and sex as covariates

Dependent Variable: Group MDD vs. HCS				
*N* = 346	Predictor:	B	Wald	*P*
Model 1	Resistin * Smoking * Sex	0.220	4.216	**0.040**
Model 2	Resistin* Smoking	0.145	7.588	**0.006**
	Resistin * Sex	-0.006	0.014	0.904
	Smoking * Sex	-0.267	0.300	0.584
Model 3	Age	0.017	3.637	0.057
	BMI	0.04	1.552	0.213
	CRP	0.017	0.81	0.368
	AUDIT sum	0.06	6.09	**0.014**
	Resistin	0.031	1.841	0.175
	Smoking	0.892	13.551	**< 0.001**
	Sex	-0.103	0.174	0.676

The table shows the valid number of subjects analyzed (N) and the results of binary regression analyses in all participants. All three models included age, BMI, CRP, AUDIT sum score, resistin, smoking status, and sex as independent variables. Model 2 additionally included the two-way interactions (Resistin × Smoking, Resistin × Sex, and Smoking × Sex). Model 1 included all aforementioned variables plus the three-way interaction (Resistin × Smoking × Sex). *P* < 0.05 in bold. HCS Healthy control subjects, MDD Major Depressive Disorder, BMI Body Mass Index, CRP C-reactive protein, AUDIT Alcohol Use Disorders Identification Test. Coding: Males = 0 vs. Females = 1; Smoking: Non-smokers: 0 vs. Smokers and Ex-smokers = 1

**Table 3:** Binary logistic regression differentiating between patient groups from healthy controls using resistin as the primary predictor and age, BMI, CRP, and AUDIT score as covariates

Dependent Variable: Group MDD vs. HCS		Age			BMI			CRP			AUDIT score			Resistin			
	*N*	B	Wald	*P*	B	Wald	*P*	B	Wald	*P*	B	Wald	*P*	B	Wald	*P*	
Male smokers and ex-smokers	84	-0.001	0.005	0.945	-0.072	0.931	0.374	0.527	1.693	**0.025**	0.110	4.924	**0.026**	0.025	0.173	0.677	
Female smokers and ex-smokers	70	-0.012	0.266	0.606	0.048	0.398	0.528	0.144	1.643	0.200	0.116	3.500	0.061	0.146	6.262	**0.012**	
Male non-smokers	93	0.042	5.425	**0.020**	-0.025	0.142	0.707	0.016	0.058	0.810	0.007	0.013	0.909	0.014	0.062	0.803	
Female non-smokers	90	0.015	0.745	0.388	0.107	1.893	0.169	-0.227	0.871	0.351	0.056	0.508	0.476	-0.067	1.490	0.222	

The table shows the valid number of subjects analyzed (N) and the results of binary logistic regression analyses in different groups, stratified by smoking status and sex. MDD Major Depressive Disorder, HCS Healthy Control Subjects, BMI Body-Mass-Index, CRP C-reactive protein, AUDIT Alcohol Use Disorders Identification Test. *P* < 0.05 in bold

**Table 4:** Linear regression to predict depression severity measured by BDI-II with resistin as primary predictor and age, BMI, CRP, AUDIT score and sex as covariates in sex stratified groups

Dependent Variable: BDI-II score		Age			BMI			CRP			AUDIT score			Resistin			
	*N*	B	T	*P*	B	T	*P*	B	T	*P*	B	T	*P*	B	T	*P*	
Male smokers and ex-smokers	54	0.088	0.872	0.388	0.092	0.224	0.824	-0.037	-0.158	0.875	0.290	1.602	0.116	0.213	0.676	0.502	
Female smokers and ex-smokers	41	-0.032	-0.228	0.821	0.134	0.361	0.720	0.061	0.406	0.687	-0.228	-1.074	0.290	-0.143	-0.524	0.603	
Male non-smokers	35	-0.031	-0.213	0.833	-0.460	-0.877	0.388	-0.340	-0.580	0.567	-0.054	-0.128	0.899	0.025	0.055	0.956	
Female non-smokers	29	-0.261	-1.480	0.152	0.442	0.484	0.633	-1.350	-0.305	0.763	0.094	0.142	0.889	-0.985	-1.628	0.117	

The table shows the valid number of subjects analyzed (N) and the results of linear regression analyses in depressed patients, stratified by smoking status and sex. BDI-II Beck Depression Inventory-II, BMI Body-Mass-Index, CRP C-reactive protein, AUDIT Alcohol Use Disorders Identification Test. *P* < 0.05 in bold

## Discussion

In this study, we examined the interplay between serum resistin levels and smoking behavior in predicting MDD risk (vs. HCS). Additional analyses assessing associations with depression severity and masculine depression symptoms were also conducted. Binary logistic regression revealed a significant three-way interaction between resistin, smoking status (smoking and ex-smoking vs. never-smoking), and sex on depression risk, alongside a significant resistin and smoking interaction. Post-hoc sex-stratified analyses showed that resistin significantly predicted MDD status among female smokers/ex-smokers, but not in other subgroups. These stratified analyses are consistent with the hypothesis that the interactive effect on MDD risk is primarily driven by female smokers and ex-smokers. Taken together, these findings suggest a potential involvement of adipokine-related mechanisms in the observed interaction pattern.

This aligns with growing evidence highlighting the role of adipokines, including resistin, in both psychiatric and somatic disorders [10]. Our results suggest that resistin may serve as a useful biomarker for targeted preventive strategies. Specifically, for the identified subpopulation of female smokers and ex-smokers, the integration of metabolic-inflammatory monitoring into psychiatric care could facilitate early identification of individuals at heightened risk. Given the observed interaction, resistin levels in this group could act as a sentinel marker, bridging the gap between lifestyle-induced inflammation and psychiatric vulnerability.

Elevated resistin in this subgroup may be driven by smoking-related mechanisms, as nicotine has been shown to induce resistin production in adipocytes [2]. However, we did not quantify the duration of ex-smoking, and resistin levels may normalize after smoking cessation. The biological plausibility primarily applies to active smokers, while it remains unclear whether the effect persists in long-term former smokers. Using a sensitivity analysis excluding former smokers, the three-way interaction terms lost statistical significance, likely reflecting reduced statistical power and loss of the intermediate exposure contrast provided by former smokers. Alternatively, higher resistin concentrations may reflect a pre-existing biological vulnerability predisposing individuals to smoking behavior and related dysregulation.

Within depressed patients, linear regression analyses found no association between resistin and depression severity (as measured by BDI-II scores) across smoking- and sex-stratified subgroups, adjusted for age, BMI, CRP, and AUDIT. This lack of correlation might be attributed to the clinical characteristics of our cohort: as the sample primarily included patients with moderate to severe depression, the resulting restriction of range may have limited the statistical power to detect a significant correlation with symptom intensity. From a broader transdiagnostic perspective, these findings suggest that resistin may function as a marker of a broader metabolic-inflammatory phenotype rather than a state-specific indicator of MDD severity. The lack of correlation could also be explained by the heterogeneity of depressive symptom presentations (e.g., somatic vs. cognitive symptoms) and the presence of comorbidities common among smokers. It remains to be explored whether this resistin-smoking interaction is unique to MDD or shared with other smoking-related mental health disorders. For instance, in schizophrenia, the role of smoking is particularly complex and involves distinct neurobiological mechanisms.

Our findings partially diverge from earlier research regarding resistin profiles in depression. Specifically, while Rahman et al., [41] reported a significant correlation between serum resistin levels and depression severity, we were unable to replicate such an association within our patient cohort. Furthermore, our study contrasts in part with the work of Małujło-Balcerska and Pietras [32] and Rahman et al., [41] in finding altered resistin profiles in depressed patients compared to healthy control groups. Instead, our results are more consistent with other studies failing to detect robust differences after controlling for confounding factors such as BMI and smoking [28]. This emphasizes the importance of adequately accounting for lifestyle-related variables when investigating inflammatory biomarkers in psychiatric populations.

As previously shown, individuals with high levels of masculine depression symptoms may be more likely to prefer alcohol or psychotropic substance use rather than seeking professional help or consulting a physician [56]. Our findings regarding the internal consistency of the MDRS-22 (α = 0.79–0.83) are largely in line with previous validation studies. For instance, Walther et al., [58] reported coefficients between 0.81 and 0.91 for most subscales, although they observed lower values for somatic symptoms (0.77) and risk-taking (0.62). In the present study, a logistic regression model predicting high vs. low masculine depression symptoms identified the AUDIT sum score, smoking and age as the predictors, whereas CRP, resistin, BMI, and sex were not significant. These findings suggest that behavioral risk factors—particularly alcohol use and smoking—play a predominant role in masculine depression symptomatology, while inflammatory markers such as resistin appear to be of limited relevance in this context when accounting for BMI and behavioral factors. Accordingly, these results do not support our initial hypothesis regarding a significant role of resistin in this setting.

The interplay between inflammatory markers like CRP and adipokines such as serum resistin is well described [4, 33]. The absence of a significant CRP effect alongside the robust interaction of resistin with smoking and sex on MDD risk in our cohort suggests that smoking-related mechanisms may drive depression vulnerability independently of baseline CRP levels, rather than through a shared inflammatory pathway.

Overall, our results suggest that circulating resistin levels interact with smoking and sex to elevate MDD risk, particularly in female smokers/ex-smokers, rather than correlating with depression severity. These findings highlight the importance of considering sex-specific and behavioral factors in studies of adipokines in depression, underscoring the complex interplay between lifestyle factors, sex, metabolic-inflammatory pathways, and psychiatric vulnerability.

### Strengths and limitations

Due to the associational design of the study, our findings do not allow conclusions regarding causality or direction. Additionally, we did not quantify the duration of ex-smoking or account for possible relapses, which should be considered in future research. Due to the lack of established MDRS-22 cut-offs, particularly in female and inpatient populations, exploratory sex-specific median splits were applied, which may limit the generalizability of the findings. Furthermore, sex was assessed only in a binary manner, which does not capture the full spectrum of gender identity and may limit the generalizability of sex-specific results. Nevertheless, the high prevalence of current and former smokers among individuals with depression underscores the relevance of our observations for this high-risk population. A major strength of the present study is the inclusion of a large, sex-balanced sample and the consideration of relevant covariates, including inflammatory markers.

## Conclusion

This study demonstrates that resistin interacts with smoking and sex to increase MDD risk, particularly in the group of female smokers and ex-smokers. Our findings suggest investigating preventive strategies in female smokers and ex-smokers with elevated resistin. This underscores the necessity of a personalized medicine approach: rather than applying generalized assumptions about smoking and inflammation, it is crucial to perform individualized risk analyses that consider the specific psychiatric diagnosis, sex-specific vulnerabilities, and the diverse nature of nicotinic effects on metabolic-inflammatory pathways. Consequently, future research should adopt a transdiagnostic and personalized medicine framework to further clarify whether these adipokine-mediated risks extend to other psychiatric populations, such as those with schizophrenia.

## Supplementary Information

Below is the link to the electronic supplementary material.

Supplementary Material 1

## Data Availability

Data are available upon request.

## Abbreviations

AUD: Alcohol Use Disorder
AUDIT: Alcohol Use Disorders Identification Test
BDI-II: Beck Depression Inventory-II
BMI: Body-Mass-Index
CRP: C-reactive protein
DSM: Diagnostic and Statistical Manual of Mental Disorders
FTND: Fagerström Test for Nicotine Dependence
GMDS: Gotland Male Depression Scale
HAMD-17: Hamilton Rating Scale for Depression-17
HCS: Healthy Control Subjects
HMD: High masculine depression scores
ICD: International Classification of Disease
IQR: Interquartile ranges
LDL: Low-density lipoprotein
LMD: Low masculine depression scores
MDRS-22: Male Depression Risk Scale
MDD: Major Depressive Disorder
