# Supplementary material for: Interaction of serum resistin and smoking behavior in predicting depression risk: a sex-sensitive analysis
Source: BMC Psychiatry. 2026 Jul 29;26:587. doi: 10.1186/s12888-026-08419-w (PMC13439829; doi:10.1186/s12888-026-08419-w)
Supplement: Supplementary file 1 [file 12888_2026_8419_MOESM1_ESM.docx]

**Supplementary Material**

**Interaction of Serum Resistin and Smoking Behavior in Predicting Depression Risk: A Sex-Sensitive Analysis**

Claudia von Zimmermann*^,1^, Christian Weinland^1^, Johannes Kornhuber^1^, Bernd Lenz^1,2,^, Christiane Mühle^1^

^1^Department of Psychiatry and Psychotherapy, Friedrich-Alexander University Erlangen-Nürnberg (FAU), Germany.

^2^Department of Addictive Behavior and Addiction Medicine, Central Institute of Mental Health (CIMH), Medical Faculty Mannheim, Heidelberg University, Germany.

*Corresponding author. E-Mail: Claudia.von.Zimmermann@uk-erlangen.de

**Supplementary Table S1** Cohort characteristics stratified by high vs. low masculine depression scores

**Supplementary Table S2** Binary logistic regression differentiating patients with high vs. low masculine depression scores using resistin as the primary predictor and age, BMI, CRP, AUDIT score, smoking, and sex as covariates

| **Supplementary Table S1** Cohort characteristics stratified by high vs. low masculine depression scores | | | | | | |
| --- | --- | --- | --- | --- | --- | --- |
|  | HMD | | LMD | | HMD vs. LMD | |
|  | N | M/F ± SD | N | M/F ± SD | χ² or t | P |
| Men | 81 | 55.6 (%) | 82 | 56.1 (%) | 0.005 | 0.944 |
| Smokers | 81 | 46.9 (%) | 81 | 29.3 (%) | 5.12 | **0.024** |
| Smokers and ex-smokers | 81 | 75.3 (%) | 82 | 43.9 (%) | 17.05 | **<0.001** |
| Ex-smokers | 81 | 28.5 (%) | 81 | 14.6 (%) | 4.41 | **0.036** |
| Age (years) | 81 | 36.41 ±14.06 | 82 | 45.67 ± 14.57 | 4.13 | **<0.001** |
| BDI-II score | 80 | 37.33 ± 10.55 | 81 | 28.74 ± 10.31 | -5.22 | **<0.001** |
| MDRS-22 score | 81 | 2.63 ± 0.79 | 82 | 1.15 ± 0.38 | -15.22 | **<0.001** |
| FTND score | 81 | 1.98 ± 2.73 | 81 | 0.96 ± 2.05 | -2.67 | **0.008** |
| AUDIT score | 80 | 9.75 ± 9.17 | 80 | 2.86 ± 3.10 | -6.74 | **<0.001** |
| CRP (mg/l) | 81 | 3.63 ± 9.04 | 82 | 3.05 ± 6.21 | -0.48 | 0.634 |
| LDL cholesterol (mg/dl) | 81 | 131.63 ± 38.32 | 82 | 136.43 ± 41.98 | 0.76 | 0.447 |
| Triglycerides (mg/dl) | 81 | 135.05 ±89.91 | 82 | 139.01 ± 80.14 | 0.30 | 0.767 |
| Resistin (ng/ml) | 81 | 14.93 ± 6.08 | 82 | 14.41 ± 5.28 | -0.59 | 0.556 |
| BMI (kg/m^2^) | 81 | 25.84 ± 4.48 | 82 | 26.34 ± 4.07 | 0.75 | 0.453 |
| Body fat (%) | 78 | 28.88 ± 9.59 | 81 | 29.59 ± 8.95 | 0.48 | 0.630 |
| Body muscle (%) | 77 | 32.41 ± 5.85 | 81 | 31.36 ± 5.24 | -1.20 | 0.234 |
| Visceral fat (%) | 78 | 7.63 ± 3.93 | 81 | 8.90 ± 4.29 | 1.95 | 0.053 |
| The table shows the valid number of subjects analyzed (N), means (M), or relative frequencies (F), standard deviation (SD), and the results of χ² and student’s t-tests. Participants with missing values for resistin were excluded from the statistical analyses. HMD High Masculine Depression scores, LMD Low Masculine Depression scores, BDI-II Beck Depression Inventory-II, MDRS-22 Male Depression Risk Scale, FTND Fagerström Test for Nicotine Dependence, AUDIT Alcohol Use Disorders Identification Test, CRP C‑reactive protein, LDL Low-density lipoprotein, BMI Body-Mass-Index. P< 0.05 in bold. | | | | | | |

| **Supplementary Table S2** Binary logistic regression differentiating patients with high vs. low masculine depression scores using resistin as the primary predictor and age, BMI, CRP, AUDIT score, smoking, and sex as covariates | | | | |
| --- | --- | --- | --- | --- |
| **Dependent Variable: Group HMD vs. LMD** | | | | |
| N= 170 | Predictor: | B | Wald | P |
|  | Age | -0.033 | 5.493 | **0.019** |
|  | BMI | -0.014 | 0.090 | 0.764 |
|  | CRP | 0.018 | 0.547 | 0.459 |
|  | AUDIT sum | 0.209 | 14.624 | **<0.001** |
|  | Smoking | 0.924 | 4.958 | **0.026** |
|  | Resistin | 0.045 | 1.645 | 0.200 |
|  | Sex | 0.303 | 0.536 | 0.464 |
| The table shows the valid number of subjects analyzed (N) and the results of binary regression analyses in depressed patients. P < 0.05 in bold. HMD High masculine depression scores, LMD Low masculine depression scores, BMI Body Mass Indes, CRP C- reactive protein, AUDIT Alcohol Use Disorders Identification Test. Coding: Males = 0 vs. Females = 1; Smoking: Non-smokers = 0 vs. Smokers and Ex-smokers = 1. | | | | |
